# Efficient clearance of Aβ protofibrils in AβPP-transgenic mice treated with a brain-penetrating bifunctional antibody

**DOI:** 10.1186/s13195-018-0377-8

**Published:** 2018-05-24

**Authors:** Stina Syvänen, Greta Hultqvist, Tobias Gustavsson, Astrid Gumucio, Hanna Laudon, Linda Söderberg, Martin Ingelsson, Lars Lannfelt, Dag Sehlin

**Affiliations:** 1 Department of Public Health and Caring Sciences/Geriatrics, Uppsala University, Rudbeck Laboratory, Dag Hammarskjölds väg 20, 75185 Uppsala, Sweden; 2 Department of Pharmaceutical biosciences, Uppsala University, Uppsala, Sweden; 3 BioArctic AB, Stockholm, Sweden

**Keywords:** Alzheimer’s disease (AD), Immunotherapy, Amyloid-β (Aβ), Oligomers, Protofibrils, Monoclonal antibody, Blood-brain barrier (BBB), Transferrin receptor (TfR)-mediated transcytosis

## Abstract

**Background:**

Amyloid-β (Aβ) immunotherapy is one of the most promising disease-modifying strategies for Alzheimer’s disease (AD). Despite recent progress targeting aggregated forms of Aβ, low antibody brain penetrance remains a challenge. In the present study, we used transferrin receptor (TfR)-mediated transcytosis to facilitate brain uptake of our previously developed Aβ protofibril-selective mAb158, with the aim of increasing the efficacy of immunotherapy directed toward soluble Aβ protofibrils.

**Methods:**

Aβ protein precursor (AβPP)-transgenic mice (tg-ArcSwe) were given a single dose of mAb158, modified for TfR-mediated transcytosis (RmAb158-scFv8D3), in comparison with an equimolar dose or a tenfold higher dose of unmodified recombinant mAb158 (RmAb158). Soluble Aβ protofibrils and total Aβ in the brain were measured by enzyme-linked immunosorbent assay (ELISA). Brain distribution of radiolabeled antibodies was visualized by positron emission tomography (PET) and ex vivo autoradiography.

**Results:**

ELISA analysis of Tris-buffered saline brain extracts demonstrated a 40% reduction of soluble Aβ protofibrils in both RmAb158-scFv8D3- and high-dose RmAb158-treated mice, whereas there was no Aβ protofibril reduction in mice treated with a low dose of RmAb158. Further, ex vivo autoradiography and PET imaging revealed different brain distribution patterns of RmAb158-scFv8D3 and RmAb158, suggesting that these antibodies may affect Aβ levels by different mechanisms.

**Conclusions:**

With a combination of biochemical and imaging analyses, this study demonstrates that antibodies engineered to be transported across the blood-brain barrier can be used to increase the efficacy of Aβ immunotherapy. This strategy may allow for decreased antibody doses and thereby reduced side effects and treatment costs.

## Background

Alzheimer’s disease (AD) is a devastating neurodegenerative disease that, despite years of effort, cannot yet be treated effectively. Immunotherapy directed against amyloid-β (Aβ), which is generally considered to drive AD pathology, appears to be the most promising strategy to modify the causal mechanisms of the progressive synapse loss and neurodegeneration [1]. A pioneering study in the late 1990s demonstrated that active vaccination cleared Aβ pathology in Aβ protein precursor (AβPP)-transgenic mice [2]. However, when tested in humans, active Aβ vaccination caused severe side effects, and the study was subsequently halted [3]. Instead, the field shifted toward passive immunization, which allows antibody properties and doses to be determined with better accuracy, potentially resulting in improved treatment efficacy and safety.

Numerous preclinical studies have shown significant effects of treatment with monoclonal Aβ antibodies in transgenic mouse models [4–7], in terms of both reduced Aβ pathology and behavioral improvement. Several of these antibodies have been further developed and studied in clinical trials, where many of them have failed to reduce cognitive decline and Aβ pathology. Although failures can probably be attributed in part to trial design and criteria for patient inclusion, one important issue is the choice of antibodies [1]. A common feature of several of the evaluated antibodies is their general Aβ-binding capacity—that is, their ability to bind and thereby be sequestered by monomeric Aβ, which is abundantly present in both blood and the central nervous system. Some of the evaluated antibodies also bind soluble forms of AβPP, which further increases the peripheral depletion of free antibody available for Aβ clearance.

The most promising immunotherapy trial data, based on the antibody aducanumab, were recently reported in *Nature* [8]. Aducanumab selectively binds to aggregated Aβ with very low affinity for Aβ monomers, which promotes specific clearance of the presumably more toxic Aβ species, without interference from monomeric Aβ or fragments of AβPP. However, this phase I trial had a limited number of participants, and the outcome must therefore be interpreted with caution.

Another challenge associated with AD immunotherapy is the limited passage of antibodies across the blood-brain barrier (BBB), which controls the transport of molecules between blood and brain. It has been reported that only 0.1% of peripherally administered antibody reaches the brain [5], and it has even been questioned whether antibodies penetrate the brain parenchyma at all [9]. The limited brain penetrance of antibodies has been addressed by various strategies for targeted delivery across the BBB, such as by using different receptors known to be present in the brain endothelium [10]. One such strategy is represented by transferrin receptor (TfR)-mediated transcytosis, which has been employed successfully to increase brain uptake of antibodies for AD [11–13]. We recently reported a novel and highly efficient brain shuttle based on a single-chain variable fragment (scFv) of the TfR-specific antibody 8D3 [14]. When recombinantly fused to the previously developed Aβ protofibril-selective antibody mAb158 [15], we obtained a bispecific antibody with low-avidity monovalent TfR binding, reducing the risk of TfR clustering on the cell surface, which could lead to degradation and downregulation of the receptor [12]. We could demonstrate a tenfold increase in brain concentration of this bispecific antibody (RmAb158-scFv8D3) in comparison with its unmodified version (RmAb158) upon intravenous administration at therapeutic doses [16].

In this study, the issues discussed above were addressed by specifically targeting soluble, aggregated Aβ with the protofibril-selective antibody mAb158, modified to be actively transported into the brain. When compared with unmodified antibody, the results not only indicated an increased therapeutic efficacy but also suggested alternative clearance mechanisms, because the modified antibody was uniformly distributed in the entire brain volume, allowing it to access and clear a larger pool of soluble Aβ. The concept of Aβ/TfR bispecific antibodies could be important for further development of AD immunotherapy, because an effective treatment could be obtained with lower antibody doses, which would reduce the risk of side effects related to high peripheral drug concentrations.

## Methods

### Animals

The AβPP-transgenic mouse model tg-ArcSwe, harboring the Arctic (*AβPP E693G*) and Swedish (*AβPP KM670/671NL*) mutations, maintained on a C57BL/6 background was used in this study. Tg-ArcSwe mice show elevated levels of soluble Aβ protofibrils at a very young age, as well as abundant and rapidly developing plaque pathology with high resemblance to human AD pathology starting around 6 months of age and increasing up to the age of 24 months [17–20]. Both males and females were used, and littermates served as wild-type control animals (wt). The animals were housed with free access to food and water in rooms with controlled temperature and humidity in an animal facility at Uppsala University.

### Antibodies and radiolabeling

The Aβ protofibril-selective antibody mAb158 binds with high avidity to soluble Aβ protofibrils, with intermediate avidity to Aβ fibrils, and with low affinity to Aβ monomers, but it does not bind to soluble fragments of AβPP [15, 21, 22]. A recombinant variant of mAb158 (RmAb158) with identical binding properties was previously recombinantly modified into a bispecific format (RmAb158-scFv8D3) for active transport across the BBB [16]. In the present study, we used RmAb158-scFv8D3, produced as previously described [23], and RmAb158 (BioArctic AB, Stockholm, Sweden). Both have been used either unmodified or radiolabeled with iodine-125 (^125^I).

Antibodies were labeled with ^125^I using direct radioiodination with the chloramine T method [24]. Briefly, 120 pmol of antibody, ^125^I solution (PerkinElmer Inc., Waltham, MA, USA) and 5 μg of chloramine T (Sigma-Aldrich, Stockholm, Sweden) were mixed in PBS to a final volume of 110 μl. The reaction was quenched after 90 seconds by addition of 10 μl of sodium metabisulfite (1 mg/ml; Sigma-Aldrich) and immediately purified with a Zeba mini desalting column, molecular weight cutoff 7 kDa (Pierce Biotechnology, Rockford, IL, USA).

### Ex vivo autoradiography

To visualize antibody distribution in the brain with ex vivo autoradiography, 18-month-old tg-ArcSwe and wt mice were given injections of 3.5 MBq [^125^I]RmAb158-scFv8D3 or 2.9 MBq [^125^I]RmAb158, equivalent to a dose of 0.20 mg of immunoglobulin G (IgG) per kilogram of body weight. Six days after injection, the mice were perfused with saline, and their brains were immediately frozen on dry ice. Coronal cryosections 50 μm thick were obtained and placed in an x-ray cassette along with ^125^I standards of known radioactivity. Positron-sensitive phosphor screens (MS [MultiSensitive]; PerkinElmer) were placed onto the samples for 5 days of exposure and then scanned at a resolution of 600 dots per inch in a Cyclone Plus imager system (PerkinElmer). The resulting digital images were converted to a false color scale (Royal) with ImageJ software (National Institutes of Health, Bethesda, MD, USA) and normalized to the standards.

### Antibody treatment and ex vivo analyses

Tg-ArcSwe mice at 14 months of age (*n* = 38) were divided into four groups of 9–11 individuals. Mice were given a single intravenous injection of PBS, a low dose of RmAb158-scFv8D3 (32 nmol/kg body weight, corresponding to 6.6 mg/kg; 1.32 mg/ml in PBS), a low dose of RmAb158 (32 nmol/kg body weight, corresponding to 5 mg/kg; 1 mg/ml in PBS), or a high dose of RmAb158 (320 nmol/kg body weight, corresponding to 50 mg/kg; 10 mg/ml in PBS). Radiolabeled protein of the same type used for the treatment and equivalent to 0.05 mg/kg IgG was mixed into the antibody solution. The specific activity was 71.2 ± 7.6 MBq/nmol for [^125^I]RmAb158-scFv8D3 and 71.9 ± 3.6 MBq/nmol for [^125^I]RmAb158. Mice were lightly sedated with isoflurane (Isoflurane Baxter®; Baxter Medical AB, Kista, Sweden), placed in a plastic holder, and intravenously administered 5 μl of antibody solution per gram of body weight. Blood samples were taken from the tail at 1, 24, and 48 hours, and a final blood sample was taken from the heart 72 hours after injection, followed by saline perfusion and isolation of the brain. Radioactivity was measured in brain and plasma with a γ-counter (1480 Wizard™; Wallac Oy, Turku, Finland), and the antibody concentrations in plasma and brain, quantified as the percentage of the injected dose (ID) per gram of tissue, were calculated as follows:

$$ \% ID\  per\ g= measured\ radioactivity\  per\  gram\ brain\ tissue\ \left( or\ plasma\right)/ injected\ radioactivity. $$

### Analyses of Aβ pathology

Following saline perfusion, the brains of mice that received injections with PBS or antibody were isolated. The right hemisphere was fixed and paraffin-embedded for IHC analysis, and the left hemisphere was homogenized in Tris-buffered saline (TBS) and formic acid (FA) to obtain extracts of soluble and total Aβ, respectively. Brain concentrations of soluble and total Aβ were measured as described previously [25]. In short, brain tissue was homogenized at a 1:5 wt/vol ratio in TBS with cOmplete protease inhibitors (Roche/Millipore Sigma, Stockholm, Sweden), then divided in two separate tubes, each mixed 1:1 with TBS and centrifuged for 1 hour at 100,000 × *g* or 16,000 × *g*, respectively. For total Aβ, the original TBS extract was mixed with concentrated FA to a concentration of 70%, followed by homogenization as described above and centrifugation at 16,000 × *g*.

#### ELISA

Aβ oligomers and protofibrils were measured in TBS soluble brain extracts with a homogeneous ELISA using 82E1 (IBL International/Tecan Trading AG, Männedorf, Switzerland) as both capture and detection antibody [26]. 82E1 is specific to the N-terminal Aβ neoepitope generated after β-secretase cleavage of AβPP. A 96-well half-area plate was coated overnight with 12.5 ng per well of 82E1, then blocked with 1% bovine serum albumin (BSA) in PBS. TBS extracts were diluted 1:10 and incubated overnight at + 4 °C, followed by detection with biotinylated 82E1 (0.25 μg/ml), streptavidin-horseradish peroxidase (SA-HRP) (1:2000; Mabtech AB, Nacka, Sweden) and K Blue Aqueous TMB Substrate (Neogen Corp., Lexington, KY, USA). For Aβ_1–40_ and Aβ_1–42_, 96-well plates were coated overnight with 100 ng per well of polyclonal rabbit anti-Aβ_40_ or anti-Aβ_42_ (Agrisera, Umeå, Sweden) and blocked with 1% BSA in PBS. FA extracts were neutralized with 2 M Tris, diluted 10,000× (Aβ_1–40_) or 2,000× (Aβ_1–42_), and incubated overnight at + 4 °C. After incubation with biotinylated 82E1 (0.25 μg/ml), signals were developed and read as described above. All dilutions were made in ELISA incubation buffer (0.1% BSA, 0.05% Tween-20 in PBS).

#### IHC

Aβ pathology was visualized with Aβ_40_ immunostaining as described previously [22] on 50-μm-thick coronal croysections adjacent to sections used for ex vivo autoradiography as well as in 5-μm sagittal sections from paraformaldehyde (PFA)-fixed, paraffin-embedded right hemispheres of mice that had undergone antibody injections at therapeutic doses. Cryosections were first fixed in 4% PFA in PBS for 20 minutes at room temperature, and paraffin sections were deparaffinized. All sections were then washed in PBS and incubated for 40 minutes in preheated citrate buffer for antigen retrieval, followed by 5-minute incubation in 70% FA, and washed under a constant flow of Milli-QH_2_O for another 5 minutes. Endogenous peroxidase activity was blocked with DAKO peroxidase block (Agilent Technologies, Kista, Sweden) for 15 minutes, followed by permeabilization with 0.4% Triton-X in PBS for 5 minutes. Unspecific binding was inhibited with DAKO block (0.25% casein in PBS), and sections were incubated overnight with 0.5 μg/ml polyclonal anti-Aβ_40_ antibody (Agrisera), then for 30 minutes with 5 μg/ml biotinylated goat antirabbit (Vector Laboratories Inc., Burlingame, CA, USA) and 30 minutes with SA-HRP (Mabtech AB). The Aβ staining was developed with NOVA RED chromogen (Vector Laboratories Inc.) for 10 minutes on a shaker, and sections were then washed in Milli-QH_2_O for 1 minute and quickly dipped first in 95% EtOH and then 99.9% EtOH. Sections were air-dried, mounted with DPX mounting medium (Sigma-Aldrich, Sweden), and analyzed with a Nikon microscope (DXM1200F; Nikon Instruments Inc., Melville, NY, USA).

### In vivo positron emission tomographic imaging after antibody pretreatment

Tg-ArcSwe mice, 18 months old and with abundant Aβ pathology, were given injections with RmAb158-scFv8D3 (32 nmol/kg body weight, *n* = 2), RmAb158 (320 nmol/kg body weight of, *n* = 2), or PBS (*n* = 2) and were then given water supplemented with 0.2% NaI to reduce thyroidal uptake of ^124^I, which can interfere with brain positron emission tomography (PET). After 3 days, all mice were given injections with 7.7 ± 0.9 MBq of [^124^I]RmAb158-scFv8D3 at a specific activity of 201 MBq/nmol antibody. Three days after injection of [^124^I]RmAb158-scFv8D3, mice were anesthetized with isoflurane (2.5% in medical air) and scanned using PET with a Triumph Trimodality PET/CT System scanner (TriFoil Imaging, Inc., Northridge, CA, USA). Anesthesia was maintained at 1.5–2.0% isoflurane, and PET data were collected in list mode for 60 minutes, followed by a computed tomographic (CT) examination for 3 minutes (field of view, 8.0 cm). PET data were reconstructed with the ordered subset expectation maximization 3D algorithm (20 iterations), and CT raw files were reconstructed using filtered back projection. All processing of PET and CT images was performed using AMIDE 1.0.434 imaging software. A T2-weighted, magnetic resonance imaging-based mouse brain atlas [27] containing outlined ROIs for hippocampus, striatum, thalamus, cortex, and cerebellum was manually aligned with the CT scan, which was then aligned with the PET scan, thus transferring the ROIs to the PET image. Quantification of PET data was presented as a concentration ratio of the radioactivity in 5 ROIs (whole brain, hippocampus, thalamus, striatum, and cortex) to that in the cerebellum, the reference region to which all PET images were normalized. This ratio is often referred to as the standardized uptake value ratio (SUVR) in PET literature and has become the main readout for Aβ imaging studies.

### Statistical analyses

Results are presented as mean ± SD. Data were analyzed with one- or two-way analysis of variance followed by Bonferroni’s or Dunnett’s post hoc test. Statistical analyses as well as plasma curve fit (one-phase decay) and AUC were calculated with Prism 6.07 software (GraphPad Software, Inc., La Jolla, CA, USA).

## Results

### Active transport improves brain uptake and distribution of mAb158

**Fig. 1:**
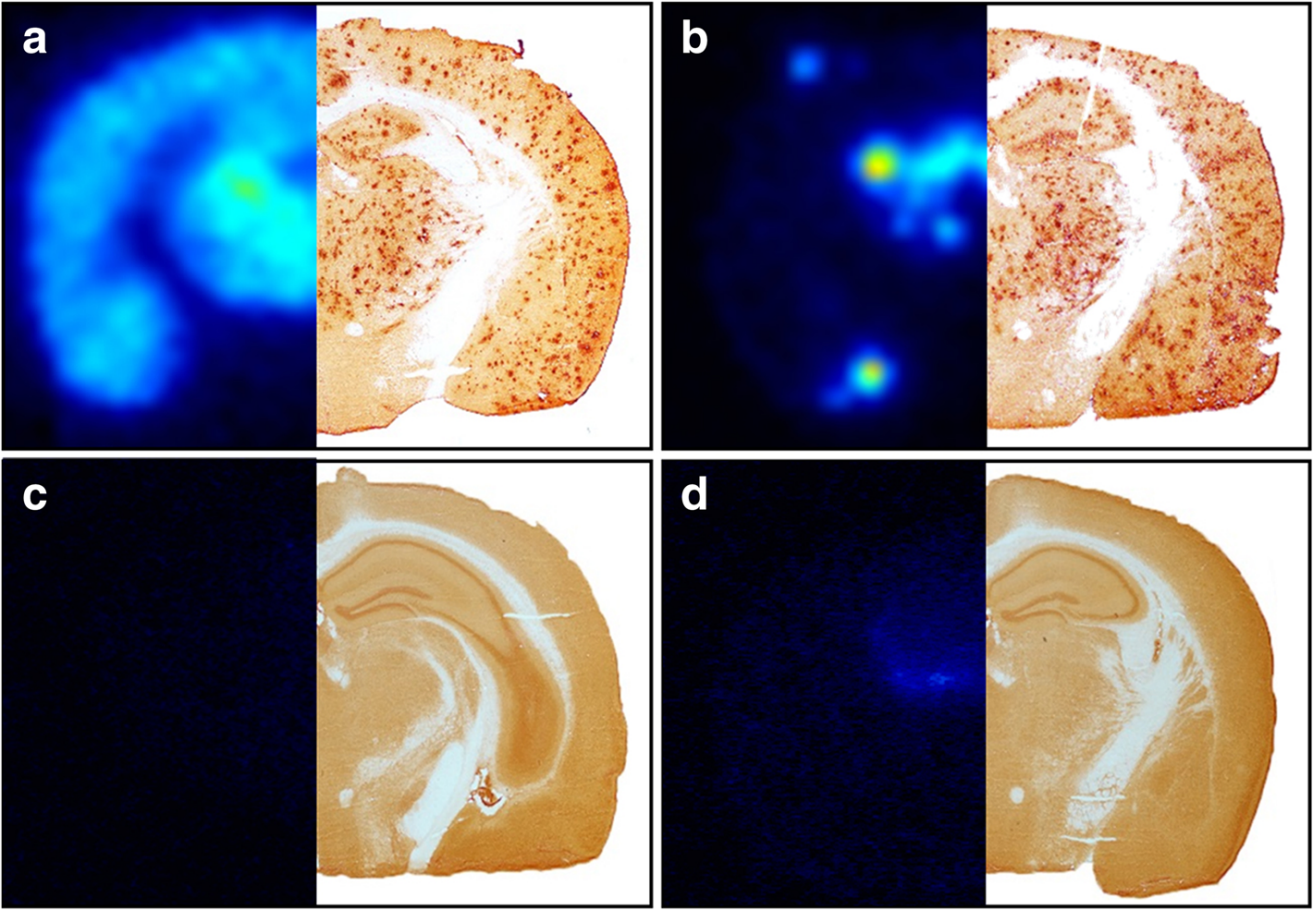
Global antibody distribution in the brain. Brain distribution in 18-month-old tg-ArcSwe mice given an injection with [^125^I]RmAb158-scFv8D3 (**a**) and [^125^I]RmAb158 (**b**), 6 days postinjection, as visualized with ex vivo autoradiography (left) in comparison with amyloid-β 40 immunostaining (right). Whereas [^125^I]RmAb158-scFv8D3 was distributed throughout the whole brain, [^125^I]RmAb158 was confined to central parts of the brain. For comparison, also wild-type animals were given an injection with [^125^I]RmAb158-scFv8D3, resulting in no signal (**c**) and with [^125^I]RmAb158, where a faint signal was detected centrally in the brain (**d**)

We have previously reported a tenfold increase in brain uptake of mAb158 recombinantly modified for active transport across the BBB by means of TfR-mediated transcytosis (RmAb158-scFv8D3), as compared with unmodified recombinant mAb158 (RmAb158) [16]. To assess whether these antibodies are also differentially distributed within the brain tissue, wt as well as 18-month-old tg-ArcSwe mice with abundant Aβ pathology were given injections with RmAb158-scFv8D3 and RmAb158 labeled with ^125^I, and the brain was isolated 6 days later. As displayed in Fig. 1, the global distribution pattern in the brain parenchyma was fundamentally different between the two antibodies. The bispecific [^125^I]RmAb158-scFv8D3 was distributed throughout the whole brain and retained in brain areas with abundant Aβ pathology, as visualized by Aβ_40_ immunostaining of an adjacent section (Fig. 1a). In contrast, [^125^I]RmAb158 was almost completely concentrated in central parts of the brain and in a few “hot spots” in the cortex (Fig. 1b). The retention of both antibodies was specific to Aβ pathology because almost no signal could be detected in wt mice (Fig. 1c and d).

Next, to study the impact of the improved brain distribution on the antibodies’ ability to reduce brain levels of soluble Aβ protofibrils, a single-dose study was conducted in 14-month-old tg-ArcSwe mice. Mice were administered a single intravenous injection of PBS, a low dose of RmAb158-scFv8D3 (32 nmol/kg), a low dose of RmAb158 (32 nmol/kg), or a high dose of RmAb158 (320 nmol/kg). These antibody doses correspond to 5 mg/kg (low dose) or 50 mg/kg (high dose) of IgG. All antibody doses were supplemented with trace amounts of ^125^I-labeled antibody of the same type to track concentrations in blood and brain. Three days after injection, the mice were perfused with saline, and their brains were extracted and analyzed.

**Fig. 2:**
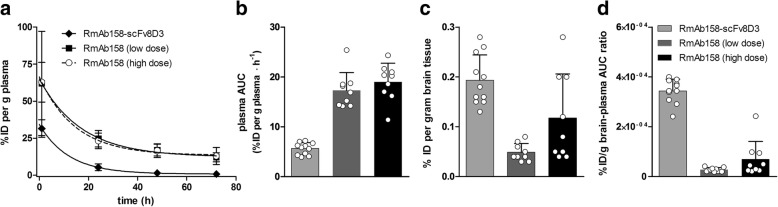
Pharmacokinetics and brain distribution. **a** Plasma pharmacokinetics of RmAb158-scFv8D3 (32 nmol/kg) and RmAb158 at two different doses (32 nmol/kg and 320 nmol/kg), expressed as the percentage of the injected dose (%ID) per gram of plasma. **b** Drug exposure calculated from the plasma AUC in (**a**). **c** Brain retention of antibodies expressed as %ID per gram of brain tissue. **d** Ratio of the antibodies’ retention in the brain to drug exposure displays their relative efficiency in entering and being retained in the brain

Compared with unmodified RmAb158, the bispecific antibody displayed a shorter half-life in blood (Fig. 2a), identical to its half-life at trace dosing [16]. This resulted in almost fourfold lower systemic drug exposure for RmAb158-scFv8D3, as demonstrated by the AUC (Fig. 2b). The lower exposure also explains why we observed a smaller difference in brain retention of the antibodies at 3 days (Fig. 2c) than the previously reported tenfold difference at 2 hours after injection [16]. However, when we adjusted for reduced exposure (i.e., calculating a ratio of brain uptake to antibody exposure), we still found a tenfold more efficient transport into the brain (Fig. 2d).

### RmAb158-scFv8D3 clears soluble Aβ protofibrils with tenfold lower dose

**Fig. 3:**
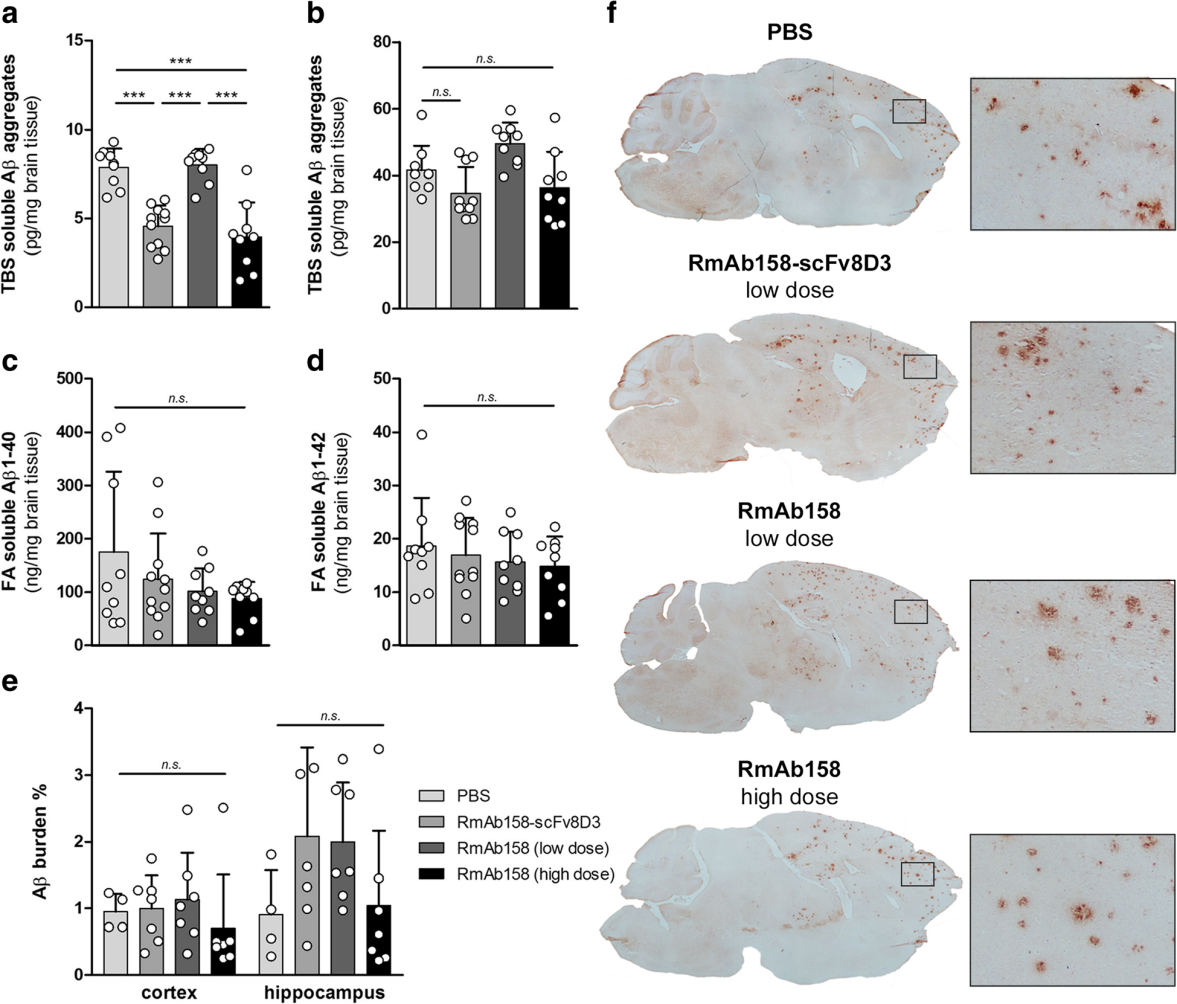
Brain levels of Tris-buffered saline (TBS) and formic acid (FA) soluble amyloid-β (Aβ). **a** Levels of soluble Aβ aggregates, measured with 82E1 homogeneous enzyme-linked immunosorbent assay in TBS brain extracts centrifuged at 100,000 × *g*. Soluble Aβ aggregates were reduced by 40% in animals dosed with RmAb158-scFv8D3 (32 nmol/kg) and a tenfold higher dose of RmAb158 (320 nmol/kg), whereas the low dose of RmAb158 (32 nmol/kg) had no effect, compared with PBS-treated animals. **b** No significant reduction of soluble Aβ aggregates was seen in TBS brain extracts centrifuged at 16,000 × *g*. Total Aβ_1–40_ (**c**) and Aβ_1–42_ (**d**) in FA soluble brain extract revealed no significant differences between the treatment groups. **e** Aβ burden, determined with Aβ_40_ IHC, showed no significant differences between the groups in either cortex or hippocampus, although the interindividual variation was high. **f** Representative images from (**e**) with magnification of the brain region marked with a *square*. All graphs display scatterplots with bars representing group mean and error bars for SD. *** *p* < 0.001 and by two-way analysis of variance followed by Bonferroni’s post hoc test. *n.s.* nonsignificant

Because soluble Aβ protofibrils are the main target species for mAb158, we measured levels of soluble Aβ aggregates in brain homogenates centrifuged at either 100,000 × *g* or 16,000 × *g*. In the most soluble fraction (100,000 × *g*) of TBS brain extracts, Aβ aggregates were decreased by more than 40% in the group of mice treated with the bispecific RmAb158-scFv8D3 (Fig. 3a) in comparison with PBS-treated mice. In contrast, an equimolar dose of RmAb158 had no effect on the levels of soluble Aβ aggregates, whereas the tenfold higher dose of RmAb158 displayed a reduction similar to that of the bispecific antibody (Fig. 3a). In the 16,000 × *g* fraction of the TBS brain extracts, no significant reduction of Aβ aggregates was observed in any of the groups (Fig. 3b). Similarly, none of the treatment groups displayed any significant effect on total Aβ levels, as measured by Aβ_1–40_ and Aβ_1–42_ ELISA in FA soluble brain extracts (Fig. 3c and d).

Total Aβ burden was also assessed with IHC and quantified as the total area covered by Aβ_40_ reactive deposits in brain sections (Fig. 3e and f). There was no significant difference between the groups, which all displayed substantial variation between mice.

### Antibody pretreatment blocks [^124^I]RmAb158-scFv8D3 PET signal

**Fig. 4:**
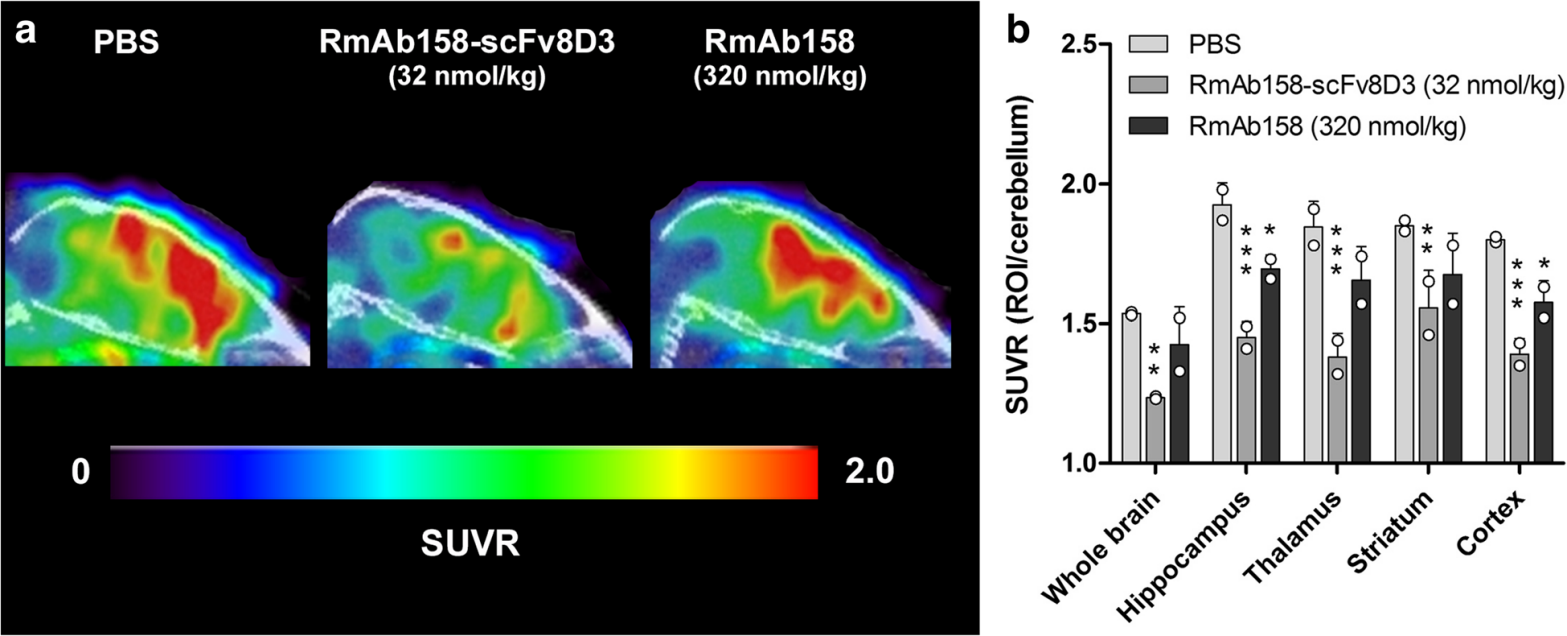
Positron emission tomographic (PET) imaging after pretreatment (blocking) with PBS, RmAb158-scFv8D3, or RmAb158. **a** Sagittal view of PET images (standardized uptake value ratio [SUVR] normalized to the reference region, the cerebellum) obtained with [^124^I]RmAb158-scFv8D3 administered to 18-month-old tg-ArcSwe mice 3 days after injection with PBS (*n* = 2), 32 nmol/kg RmAb158-scFv8D3 (*n* = 2), or 320 nmol/kg RmAb158 (*n* = 2). **b** Quantification of SUVR (ROI/cerebellum) from PET images in (**a**) for whole brain, hippocampus, thalamus, striatum, and cortex. Graph displays scatterplot with bars representing group mean and error bars for SD. * *p* < 0.05, ** *p* < 0.01, and *** p < 0.001 in comparison with the PBS group by two-way analysis of variance and Dunnett’s post hoc test

To further elucidate the difference in brain distribution of RmAb158-scFv8D3 and RmAb158, a PET experiment was conducted in which tg-ArcSwe mice were pretreated with a low dose of RmAb158-scFv8D3 (32 nmol/kg), a high dose of RmAb158 (320 nmol/kg), or PBS 3 days before PET imaging with [^124^I]RmAb158-scFv8D3 (Fig. 4a). The resulting PET images therefore represent the brain distribution of [^124^I]RmAb158-scFv8D3 after in vivo “competition” for Aβ binding sites with the preinjected antibody (i.e., RmAb158-scFv8D3 or RmAb158). Mice pretreated with RmAb158-scFv8D3 showed significantly lower SUVRs in all investigated regions (*p* < 0.01–0.001) in comparison with PBS injected mice. RmAb158-pretreated mice showed reduced SUVRs compared with PBS mice, but to a lesser extent, and the decrease was significant only in the hippocampus (*p* < 0.05) and the cortex (*p* < 0.05) (Fig. 4b).

## Discussion

We have previously shown that mAb158 can efficiently and selectively reduce brain levels of Aβ protofibrils in the tg-ArcSwe mouse model and, if given at an early stage of Aβ pathology, can prevent accumulation of Aβ plaques [4]. A humanized version of this antibody, BAN2401 [28], is currently being evaluated in a phase IIb clinical trial [29]. Similar to aducanumab, which recently showed promising results in a small trial, mAb158 binds with high selectivity to aggregated Aβ and has a very low affinity for monomeric Aβ. This selectivity could be key to successfully eliminating the pathogenic species of Aβ while leaving the abundant, and possibly physiologically relevant, Aβ monomers intact. Further, mAb158 binds with moderate affinity to Aβ fibrils, which could decrease the risk of side effects related to antibody interactions with fibrillar Aβ deposits in vessel walls. Such interactions may cause microbleeding and amyloid-related imaging abnormalities, which are frequently observed side effects in AD immunotherapy trials. In this respect, mAb158/BAN2401 may differ from aducanumab, which is reported to bind fibrillar Aβ with high affinity [8].

In the present study, we have demonstrated that a single injection of RmAb158, given at a high dose (320 nmol/kg), can efficiently reduce brain levels of soluble Aβ protofibrils, whereas a tenfold lower dose had no such effect. However, using RmAb158-scFv8D3, a bispecific variant of the antibody engineered to enable receptor-mediated transcytosis across the BBB [16], we obtained a similar Aβ reduction with only 10% of the dose (Fig. 3). This result is in itself promising because it suggests that antibody doses can be significantly reduced while retaining the treatment effect and also that a substantial reduction of soluble Aβ aggregates can be obtained only 3 days after a single antibody injection. Moreover, on one hand, the bispecific RmAb158-scFv8D3 was distributed to the brain parenchyma in a uniform pattern and was retained in brain regions with abundant Aβ pathology. This distribution pattern is in line with our previous finding that mAb158, transcytosed into the brain by chemical conjugation to 8D3, and accumulated around parenchymal plaques [21], a site where Aβ oligomerization has been reported to occur [30]. RmAb158, on the other hand, was distributed mainly to the central periventricular areas, with a few “hot spots” in cortical areas (Fig. 1), in agreement with an enriched signal in the choroid plexus of tg-ArcSwe mice 3 days after administration of radiolabeled mAb158 [22]. These findings, together with the reported observation that peripherally injected antibodies appear in the CSF, support the notion that antibodies may enter the brain by crossing the blood-CSF barrier rather than the BBB, resulting in a limited parenchymal distribution [9]. The few hot spots of RmAb158 deposition in the cortex could potentially be explained by a locally damaged BBB, resulting in antibody leakage into the brain at those sites. This phenomenon is likely concentration-dependent and could be the reason that some animals in the RmAb158 high-dose group showed increased antibody retention in the brain (Fig. 2c).

Hence, both the unmodified RmAb158 and the bispecific RmAb158-scFv8D3 have the ability to reduce brain levels of soluble Aβ aggregates, but RmAb158-scFv8D3 seems to have a tenfold higher efficiency in eliminating its target from the brain. However, the brain retention results indicate that 3 days after injection, there was only a two- to fourfold difference between the antibody variants in relative concentrations associated with the brain (Fig. 2c). This suggests that not only the amount of antibody retained in the brain but also its regional distribution (Fig. 1) is important for Aβ clearance. RmAb158-scFv8D3 treatment would thus efficiently target soluble Aβ aggregates in all areas of the brain, whereas RmAb158 may sequester Aβ predominantly at specific sites.

To investigate this question further, old tg-ArcSwe mice were given the same therapeutic doses of RmAb158-scFv8D3 (32 nmol/kg) or RmAb158 (320 nmol/kg) that had proven to efficiently reduce brain levels of soluble Aβ aggregates. Subsequent PET imaging with [^124^I]RmAb158-scFv8D3, which specifically visualizes Aβ pathology in vivo [16], allowed us to investigate the in vivo distribution of [^124^I]RmAb158-scFv8D3 in the presence of preinjected antibody, which, if bound to intraparenchymal Aβ, would reduce the [^124^I]RmAb158-scFv8D3 PET signal. This experiment clearly showed that the [^124^I]RmAb158-scFv8D3 PET signal was substantially blocked in all selected brain regions in mice given the relatively low dose of RmAb158-scFv8D3, whereas the signal in mice given a tenfold higher dose of RmAb158 was blocked to a lesser degree and only in two brain regions. This result, which is based on 3D quantification of brain concentrations of [^124^I]RmAb158-scFv8D3, confirms that RmAb158 is not localized to the same areas as RmAb158-scFv8D3 and supports the idea that RmAb158-scFv8D3 and RmAb158 clear soluble Aβ aggregates at different sites and possibly through different mechanisms. It also implies that RmAb158-scFv8D3, which is distributed to the entire volume of the brain, has access to a much larger pool of soluble Aβ that it can eliminate, further emphasizing the benefit of employing a receptor-mediated transcytosis strategy for Aβ immunotherapy.

The blood pharmacokinetics of RmAb158-scFv8D3 seem to be dose-independent, because the blood concentration curve in this study (Fig. 2a) was identical to that of a previous study, where the antibody was given at tracer dose [16]. On one hand, the bispecific antibody’s rapid elimination from blood could be a limitation because it results in decreased drug exposure (as represented by the blood AUC in Fig. 2a). On the other hand, low peripheral antibody concentrations likely reduce side effects. Moreover, we have previously demonstrated that when entered into the brain of old tg-ArcSwe mice, the antibody is retained in high concentrations over a period of at least 10 days, with a half-life in brain substantially longer than that in blood [16]. Thus, if dosed regularly, the brain antibody concentrations may be kept high enough to maintain a therapeutic effect over time. Studies conducted over a longer period of time are needed to investigate this further. Such studies will also have to elucidate the effect of RmAb158-scFv8D3 treatment on plaque pathology as well as markers associated with activated microglia and reactive astrocytes that accompany the plaque pathology both in AD and in this mouse model [31–33]. In the present study, with such a short treatment period and only a single antibody injection, we could see a reduction of the most soluble Aβ aggregates (i.e., in the brain extract centrifuged at 100,000 × *g*), whereas no significant effect was seen in the 16,000 × *g* extract (Fig. 3a and b). The latter fraction probably also contains aggregates that can still be considered soluble, but these aggregates are less diffusible and thus less prone to be cleared by a single dose of antibody. It can be speculated that with repeated dosing, the bispecific antibody, covering a larger volume of the brain parenchyma, is more likely than the unmodified RmAb158 to have an effect on this type of pathology. In this study, an effect on plaque pathology was not expected. Indeed, IHC quantification of Aβ pathology did not show any significant differences between groups, although there was a substantial variation between animals. These results were also confirmed by ELISA quantification of Aβ_1–40_ and Aβ_1–42_ in FA soluble brain extract, which represents the total burden of Aβ, including plaques, in the whole volume of the brain.

## Conclusions

By combining Aβ immunotherapy and in vivo PET imaging, this study demonstrates that TfR-mediated transcytosis can markedly improve brain uptake and distribution of the Aβ protofibril-selective antibody mAb158, leading to an increased target engagement in the brain of transgenic mice (tg-ArcSwe) with AD-like Aβ pathology, probably through a mechanism that is different from traditional Aβ immunotherapy. This strategy increases therapeutic efficacy and may allow for decreased antibody doses to be administered, thereby reducing side effects and treatment costs.

## References

[1] Lannfelt L, Möller C, Basun H, Osswald G, Sehlin D, Satlin A, Logovinsky V, Gellerfors P (2014). Perspectives on future Alzheimer therapies: amyloid-β protofibrils - a new target for immunotherapy with BAN2401 in Alzheimer’s disease. Alzheimers Res Ther.

[2] Schenk D, Barbour R, Dunn W, Gordon G, Grajeda H, Guido T, Hu K, Huang J, Johnson-Wood K, Khan K (1999). Immunization with amyloid-β attenuates Alzheimer-disease-like pathology in the PDAPP mouse. Nature.

[3] Orgogozo JM, Gilman S, Dartigues JF, Laurent B, Puel M, Kirby LC, Jouanny P, Dubois B, Eisner L, Flitman S (2003). Subacute meningoencephalitis in a subset of patients with AD after Aβ42 immunization. Neurology.

[4] Lord A, Gumucio A, Englund H, Sehlin D, Sundquist VS, Söderberg L, Möller C, Gellerfors P, Lannfelt L, Pettersson FE, Nilsson LN (2009). An amyloid-β protofibril-selective antibody prevents amyloid formation in a mouse model of Alzheimer’s disease. Neurobiol Dis.

[5] Bard F, Cannon C, Barbour R, Burke RL, Games D, Grajeda H, Guido T, Hu K, Huang J, Johnson-Wood K (2000). Peripherally administered antibodies against amyloid β-peptide enter the central nervous system and reduce pathology in a mouse model of Alzheimer disease. Nat Med.

[6] DeMattos RB, Bales KR, Cummins DJ, Dodart JC, Paul SM, Holtzman DM (2001). Peripheral anti-Aβ antibody alters CNS and plasma Aβ clearance and decreases brain Aβ burden in a mouse model of Alzheimer’s disease. Proc Natl Acad Sci U S A.

[7] Tucker S, Möller C, Tegerstedt K, Lord A, Laudon H, Sjödahl J, Söderberg L, Spens E, Sahlin C, Waara ER (2015). The murine version of BAN2401 (mAb158) selectively reduces amyloid-β protofibrils in brain and cerebrospinal fluid of tg-ArcSwe mice. J Alzheimers Dis.

[8] Sevigny J, Chiao P, Bussiere T, Weinreb PH, Williams L, Maier M, Dunstan R, Salloway S, Chen T, Ling Y (2016). The antibody aducanumab reduces Aβ plaques in Alzheimer’s disease. Nature.

[9] Pardridge WM (2016). CSF, blood-brain barrier, and brain drug delivery. Expert Opin Drug Deliv.

[10] Pardridge WM (2016). Re-engineering therapeutic antibodies for Alzheimer’s disease as blood-brain barrier penetrating bi-specific antibodies. Expert Opin Biol Ther.

[11] Yu YJ, Zhang Y, Kenrick M, Hoyte K, Luk W, Lu Y, Atwal J, Elliott JM, Prabhu S, Watts RJ, Dennis MS (2011). Boosting brain uptake of a therapeutic antibody by reducing its affinity for a transcytosis target. Sci Transl Med.

[12] Niewoehner J, Bohrmann B, Collin L, Urich E, Sade H, Maier P, Rueger P, Stracke JO, Lau W, Tissot AC (2014). Increased brain penetration and potency of a therapeutic antibody using a monovalent molecular shuttle. Neuron.

[13] Sumbria RK, Hui EK, Lu JZ, Boado RJ, Pardridge WM (2013). Disaggregation of amyloid plaque in brain of Alzheimer’s disease transgenic mice with daily subcutaneous administration of a tetravalent bispecific antibody that targets the transferrin receptor and the Aβ amyloid peptide. Mol Pharm.

[14] Kissel K, Hamm S, Schulz M, Vecchi A, Garlanda C, Engelhardt B (1998). Immunohistochemical localization of the murine transferrin receptor (TfR) on blood-tissue barriers using a novel anti-TfR monoclonal antibody. Histochem Cell Biol.

[15] Englund H, Sehlin D, Johansson AS, Nilsson LN, Gellerfors P, Paulie S, Lannfelt L, Pettersson FE (2007). Sensitive ELISA detection of amyloid-β protofibrils in biological samples. J Neurochem.

[16] Hultqvist G, Syvänen S, Fang XT, Lannfelt L, Sehlin D (2017). Bivalent brain shuttle increases antibody uptake by monovalent binding to the transferrin receptor. Theranostics.

[17] Lord A, Kalimo H, Eckman C, Zhang XQ, Lannfelt L, Nilsson LN (2006). The Arctic Alzheimer mutation facilitates early intraneuronal Aβ aggregation and senile plaque formation in transgenic mice. Neurobiol Aging.

[18] Lord A, Englund H, Söderberg L, Tucker S, Clausen F, Hillered L, Gordon M, Morgan D, Lannfelt L, Pettersson FE, Nilsson LN (2009). Amyloid-β protofibril levels correlate with spatial learning in Arctic Alzheimer’s disease transgenic mice. FEBS J.

[19] Philipson O, Hammarström P, Nilsson KP, Portelius E, Olofsson T, Ingelsson M, Hyman BT, Blennow K, Lannfelt L, Kalimo H, Nilsson LN (2009). A highly insoluble state of Aβ similar to that of Alzheimer’s disease brain is found in Arctic APP transgenic mice. Neurobiol Aging.

[20] Sehlin D, Fang XT, Meier SR, Jansson M, Syvänen S. Pharmacokinetics, biodistribution and brain retention of a bispecific antibody-based PET radioligand for imaging of amyloid-β. Sci Rep. 2017;7(1):17254. 10.1038/s41598-017-17358-2 PMC5722892 29222502

[21] Sehlin D, Fang XT, Cato L, Antoni G, Lannfelt L, Syvänen S (2016). Antibody-based PET imaging of amyloid β in mouse models of Alzheimer’s disease. Nat Commun.

[22] Magnusson K, Sehlin D, Syvänen S, Svedberg MM, Philipson O, Söderberg L, Tegerstedt K, Holmquist M, Gellerfors P, Tolmachev V (2013). Specific uptake of an amyloid-β protofibril-binding antibody-tracer in AβPP transgenic mouse brain. J Alzheimers Dis.

[23] Fang XT, Sehlin D, Lannfelt L, Syvänen S, Hultqvist G (2017). Efficient and inexpensive transient expression of multispecific multivalent antibodies in Expi293 cells. Biol Proced Online.

[24] Greenwood FC, Hunter WM, Glover JS (1963). The preparation of I-131-labelled human growth hormone of high specific radioactivity. Biochem J.

[25] Syvänen S, Fang XT, Hultqvist G, Meier SR, Lannfelt L, Sehlin D (2017). A bispecific Tribody PET radioligand for visualization of amyloid-β protofibrils - a new concept for neuroimaging. Neuroimage.

[26] Xia W, Yang T, Shankar G, Smith IM, Shen Y, Walsh DM, Selkoe DJ (2009). A specific enzyme-linked immunosorbent assay for measuring β-amyloid protein oligomers in human plasma and brain tissue of patients with Alzheimer disease. Arch Neurol.

[27] Ma Y, Hof PR, Grant SC, Blackband SJ, Bennett R, Slatest L, McGuigan MD, Benveniste H (2005). A three-dimensional digital atlas database of the adult C57BL/6J mouse brain by magnetic resonance microscopy. Neuroscience.

[28] Logovinsky V, Satlin A, Lai R, Swanson C, Kaplow J, Osswald G, Basun H, Lannfelt L (2016). Safety and tolerability of BAN2401 - a clinical study in Alzheimer’s disease with a protofibril selective Aβ antibody. Alzheimers Res Ther.

[29] Satlin A, Wang J, Logovinsky V, Berry S, Swanson C, Dhadda S, Berry DA (2016). Design of a Bayesian adaptive phase 2 proof-of-concept trial for BAN2401, a putative disease-modifying monoclonal antibody for the treatment of Alzheimer’s disease. Alzheimers Dement (N Y).

[30] Koffie RM, Meyer-Luehmann M, Hashimoto T, Adams KW, Mielke ML, Garcia-Alloza M, Micheva KD, Smith SJ, Kim ML, Lee VM (2009). Oligomeric amyloid β associates with postsynaptic densities and correlates with excitatory synapse loss near senile plaques. Proc Natl Acad Sci U S A.

[31] Söllvander S, Nikitidou E, Brolin R, Söderberg L, Sehlin D, Lannfelt L, Erlandsson A (2016). Accumulation of amyloid-β by astrocytes result in enlarged endosomes and microvesicle-induced apoptosis of neurons. Mol Neurodegener.

[32] Olsen M, Aguilar X, Sehlin D, et al. Mol Imaging Biol Astroglial responses to amyloid-β pathology progression in a mouse model of Alzheimer’s disease. Mol Imaging Biol; 2018. 10.1007/s11307-017-1153-z. 10.1007/s11307-017-1153-z 29297157

[33] Söllvander S, Nikitidou E, Gallasch L, Zysk M, Söderberg L, Sehlin D, Lannfelt L, Erlandsson A. The Aβ protofibril selective antibody mAb158 prevents accumulation of Aβ in astrocytes and rescues neurons from Aβ-induced cell death. J Neuroinflammation. 2018;15(1):98. 10.1186/s12974-018-1134-4 PMC5875007 29592816

